# The incidence of peripartum invasive group A streptococcal infections and association with illicit drug use

**DOI:** 10.1016/j.xagr.2025.100534

**Published:** 2025-06-11

**Authors:** Elizabeth Wagman, Matthew Pettengill, Rupsa C. Boelig, Sarah Boudova

**Affiliations:** Sidney Kimmel Medical College, Thomas Jefferson University, Philadelphia, PA; Department of Pathology and Genomic Medicine, Thomas Jefferson University, Philadelphia, PA; Sidney Kimmel Medical College, Department of Obstetrics and Gynecology, Division of Maternal-Fetal Medicine, Thomas Jefferson University, Philadelphia, PA

## Objective

Group A streptococcal (GAS) infection ranges from mild infections to severe conditions like pneumonia, sepsis, and necrotizing fasciitis.^1^ Pregnancy increases the risk of adverse outcomes, including an 89-fold higher risk of puerperal sepsis.^2^ The incidence of invasive GAS (iGAS) appears to be rising, particularly in the pediatric population in the U.S. following the COVID-19 pandemic, though the impact on pregnant individuals is unclear.^2^^,^^3^ iGAS in pregnancy remains underexplored, with studies largely conducted before the pandemic.^1^ We aimed to determine the incidence of peripartum iGAS in a major Philadelphia health system and to identify associated risk factors, particularly injection drug use (IDU), timing, and outcomes.

## Study design

We performed a retrospective chart review of all iGAS case in the Thomas Jefferson University Health System Pathology records between April 1, 2017 (date the electronic medical record system was implemented), and June 30, 2023. Peripartum iGAS incidence was calculated by dividing cases in pregnant persons by total deliveries (n = 17,277). The study was approved by the Thomas Jefferson University IRB. Descriptive statistics were performed using Excel.

## Results

In our study period, 17 cases of peripartum iGAS were reported, with an incidence of 0.98 cases per 1,000 births. Of these, 12 (70.6%) were antepartum, 3 (17.6%) intrapartum, and 2 (11.8%) postpartum. One 17w5d miscarriage secondary to placental abruption associated with IDU occurred (Table 1, case 9). No other maternal, fetal, or neonatal mortality was reported. One patient (Table 1, case 14) had children at home, and 13 (76.5%) reported IDU (polysubstance use). Infections were often polymicrobial (n = 11, 64.7%), typically involving methicillin-resistant *Staphylococcus aureus* (n = 7, 41.2%). One patient (Table 1, case 13) had iGAS endometritis. Six (35.3%) patients needed surgical wound treatment. Of the patients who had follow-up care within three weeks of infection, no nonsuppurative complications of iGAS were recorded. The highest iGAS incidence was in 2018 (Supplemental Figure 1). Rates did not increase above baseline following the pandemic.

**Table 1:** Patient demographics, clinical features, risk factors, and surgical management of peripartum iGAS cases in a major health system in Philadelphia from April 1, 2017, to June 30, 2023

Case number	Year	Patient age	Gravidity and parity	Gestational age at iGAS diagnosis^a^	iGAS infection collection site	Polymicrobial infection	IV antibiotic use (days)	Surgical management	ICU admission for management of iGAS	Pregnancy outcome	Risk factors noted in EMR
1	2018	31	G7P2042	Unknown	Sputum	No	None	None	No	Lost to follow-up	Sick contact, IDU
2	2018	32	G3P0111	36w2d^b^	Arm cellulitis	Yes	None	None	No	SVD at home	IDU
3	2018	23	G2P1011	36w5d	Elbow abscess	Yes	29	Abscess I&D, wound debridement	No	CS	IDU
4	2018	27	G3P2103	19w1d	Arm abscess	Yes	None	Abscess I&D, wound debridement	No	Lost to follow-up	IDU
5	2018	29	G3P0020	6w1d	Arm abscess	Yes	1	None	No	SVD	IDU
6	2019	33	G13P7057	34w3d	Neck abscess	Yes	1	None	No	Lost to follow-up	IDU
7	2019	40	G4P3013	PPD #1	Urine, sputum	No	1	None	No	SVD	None
8	2019	24	G2P0010	Unknown	Tonsillar abscess	No	3	Abscess I&D at bedside	No	Lost to follow-up	None
9	2019	31	G1P0	17w5d^b^	Urine	No	None	None	No	Spontaneous abortion	IDU
10	2020	35	G2P2002	19w3d	Leg cellulitis	Yes	None	None	No	C-section	None
11	2021	31	G2P1102	33w4d	Multiple abscesses	Yes	8	Abscess I&D, wound debridement	Yes	C-section	IDU
12	2021	30	G6P1041	16w3d	Neck abscess	Yes	17	Abscess I&D, wound debridement	No	SVD	IDU
13	2021	36	G4P3104	PPD #0	Endometritis, bacteremia	No	9	None	Yes	SVD	IDU
14	2022	28	G4P2113	36w5d^b^	Blood - Bacteremia	No	4	None	No	CS	Children at home
15	2022	30	G3P1112	34w4d	Multiple abscesses	Yes	5	None	No	CS	IDU
16	2022	27	G3P0110	21w0d	Periorbital abscess	Yes	3	Abscess I&D, wound debridement	No	Lost to follow-up	IDU
17	2023	30	G3P0010	5w6d	Burn	Yes	9	Skin graft over full-thickness burn	No	Medication abortion	IDU

*CS*, cesarean section; *EMR*, electronic medical record; *I&D,* incision and drainage; *ICU,* intensive care unit; *IDU*, illicit drug use; *iGAS*, invasive group A streptococcus; *IV*, intravenous; *PPD#,* post-partum day number; *SVD,* spontaneous vaginal delivery.

a For postpartum patients, postpartum day number (PPD#) is noted for presentation of iGAS infection.

b Indicates intrapartum iGAS infection timing.

## Conclusion

We observed an 8-fold higher incidence of peripartum iGAS infection in our population compared to the global incidence of 0.12 cases per 1000 births.^1^ This may be partially due to high rates of IDU in Philadelphia. Elevated IDU rates among iGAS patients align with trends in non-pregnant populations locally and nationally.^4^ We observed the highest rates in 2018, a year when hospitalizations for IDU-related endocarditis peaked in Philadelphia.^5^

Young children at home is a known risk factor for iGAS in pregnancy.^2^ One patient in our study had this risk factor, although records typically did not comment on it, so this was not systematically queried. Unlike trends observed in pediatrics, we did not see higher rates of iGAS infections following the pandemic, suggesting that children may not be the primary risk factor for iGAS in our population.^3^

Study strengths include detailed case examination and post-COVID-19 pandemic data. Limitations include a small sample size, loss to follow-up, and inability to assess IDU type, including the potential role of xylazine, an iGAS risk factor.^5^ National studies on peripartum iGAS are warranted, including investigating GAS screening in high-risk populations and evaluation of additional risk factors, such as immunosuppression in pregnancy. In the meantime, physicians should maintain a high index of suspicion for iGAS in pregnant patients with IDU, even in those without signs of GAS endometritis, as they may present atypically in the clinical setting. Future research is essential to better understand and prevent peripartum iGAS.

## CRediT authorship contribution statement

**Elizabeth Wagman:** Writing – original draft. **Matthew Pettengill:** Writing – review & editing. **Rupsa C. Boelig:** Writing – review & editing. **Sarah Boudova:** Writing – review & editing.

## Appendix Supplementary materials

Image, application 1

## Footnotes

Supplementary material associated with this article can be found, in the online version, at doi:10.1016/j.xagr.2025.100534.
