# Supplementary material for: The incidence of peripartum invasive group A streptococcal infections and association with illicit drug use
Source: AJOG Glob Rep. 2025 Jun 11;5(3):100534. doi: 10.1016/j.xagr.2025.100534 (PMC12271608; doi:10.1016/j.xagr.2025.100534)
Supplement: Supplementary file 1 [file mmc1.docx]

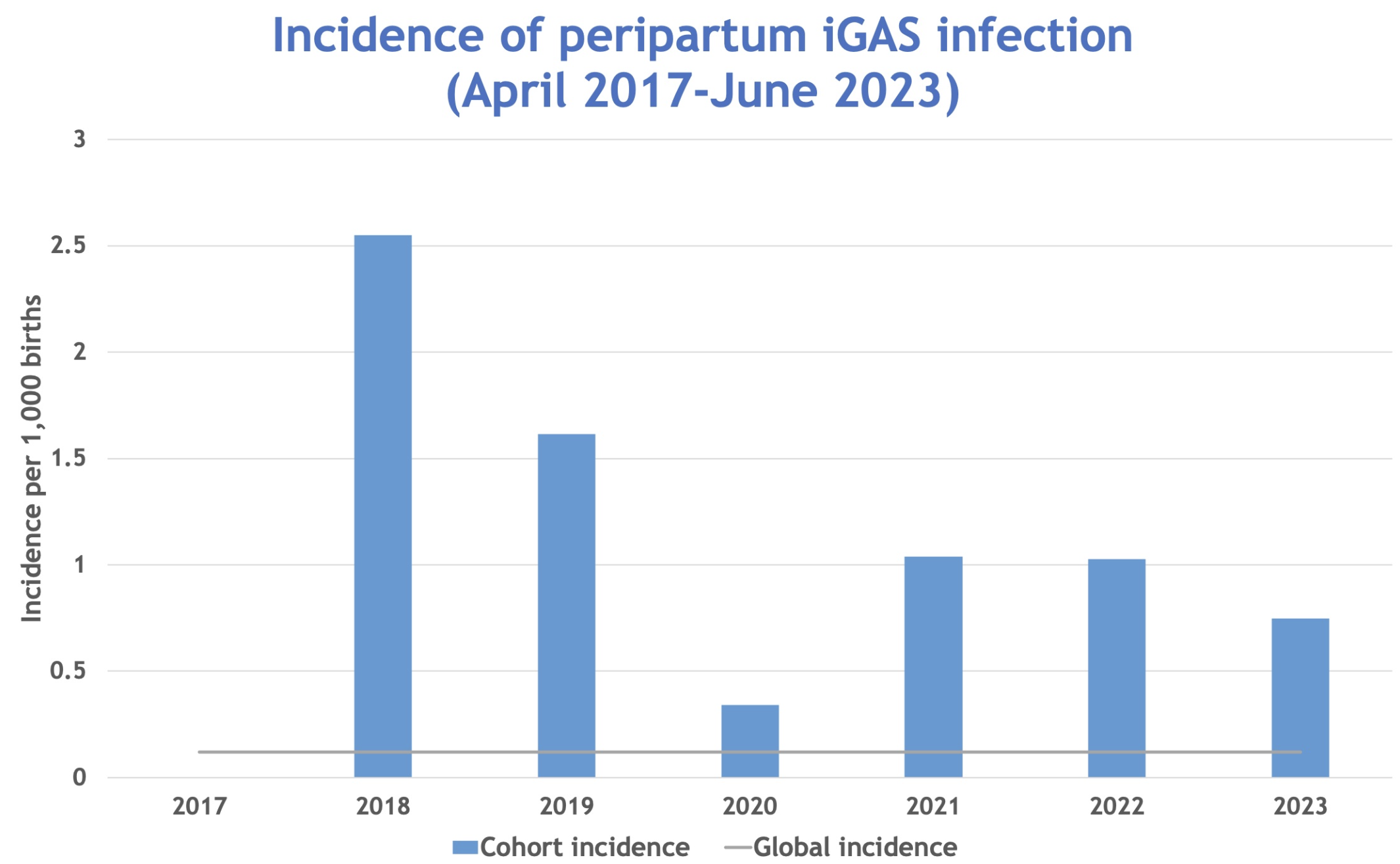


Supplemental figure 1. Incidence of invasive Group A Streptococcus (iGAS) infection in a major health system in Philadelphia from April 1, 2017, to June 30, 2023, is shown in blue bars. The estimated global incidence of 0.12 per 1,000 births is indicated with a grey line.
